# The ERAD Inhibitor Eeyarestatin I Is a Bifunctional Compound with a Membrane-Binding Domain and a p97/VCP Inhibitory Group

**DOI:** 10.1371/journal.pone.0015479

**Published:** 2010-11-12

**Authors:** Qiuyan Wang, Bidhan A. Shinkre, Jin-gu Lee, Marc A. Weniger, Yanfen Liu, Weiping Chen, Adrian Wiestner, William C. Trenkle, Yihong Ye

**Affiliations:** 1 Laboratory of Molecular Biology, National Institute of Diabetes and Digestive and Kidney Diseases, National Institutes of Health, Bethesda, Maryland, United States of America; 2 Hematology Branch, National Heart, Lung, and Blood Institute, National Institutes of Health, Bethesda, Maryland, United States of America; 3 Laboratory of Cell Biochemistry and Biology, National Institute of Diabetes and Digestive and Kidney Diseases, National Institutes of Health, Bethesda, Maryland, United States of America; 4 The Genomics Core Laboratory, National Institute of Diabetes and Digestive and Kidney Diseases, National Institutes of Health, Bethesda, Maryland, United States of America; Griffith University, Australia

## Abstract

**Background:**

Protein homeostasis in the endoplasmic reticulum (ER) has recently emerged as a therapeutic target for cancer treatment. Disruption of ER homeostasis results in ER stress, which is a major cause of cell death in cells exposed to the proteasome inhibitor Bortezomib, an anti-cancer drug approved for treatment of multiple myeloma and Mantle cell lymphoma. We recently reported that the ERAD inhibitor Eeyarestatin I (EerI) also disturbs ER homeostasis and has anti-cancer activities resembling that of Bortezomib.

**Methodology and Principal Findings:**

Here we developed *in vitro* binding and cell-based functional assays to demonstrate that a nitrofuran-containing (NFC) group in EerI is the functional domain responsible for the cytotoxicity. Using both SPR and pull down assays, we show that EerI directly binds the p97 ATPase, an essential component of the ERAD machinery, via the NFC domain. An aromatic domain in EerI, although not required for p97 interaction, can localize EerI to the ER membrane, which improves its target specificity. Substitution of the aromatic module with another benzene-containing domain that maintains membrane localization generates a structurally distinct compound that nonetheless has similar biologic activities as EerI.

**Conclusions and Significance:**

Our findings reveal a class of bifunctional chemical agents that can preferentially inhibit membrane-bound p97 to disrupt ER homeostasis and to induce tumor cell death. These results also suggest that the AAA ATPase p97 may be a potential drug target for cancer therapeutics.

## Introduction

The endoplasmic reticulum (ER) is a major site of protein folding and assembly in eukaryotic cells. Although many chaperones are present in the ER to assist proteins in folding, misfolded polypeptides are frequently produced, disturbing ER homeostasis. The accumulation of misfolded proteins in ER triggers ER stress, a condition that activates several signaling pathways collectively termed unfolded protein response (UPR) [1], [2]. As a major mechanism that adapts cells to ER stress, UPR promotes the elimination of misfolded proteins from the ER. This is critical for cell vitality, particularly for those carrying high secretory loads. Moreover, UPR is often activated in tumor tissues due to the hypoxia condition under which cancer cells are grown [3], [4] and this mild UPR activation is believed to promote cancer progression as it helps to improve ER fitness and overall cell vitality [5], [6], [7], [8]. On the other hand, if UPR fails to rectify the folding problem as often seen in damaged or aged tissues or cells overexposed to pharmacological ER stressors, misfolded proteins can accumulate beyond a reversible point. This causes an irreversible disruption of ER homeostasis [9]. Signaling processes associated with programmed cell death are then activated [10], [11], [12], [13].

Healthy cells maintain ER homeostasis by delicately monitoring the load of proteins into the ER, fine-tuning the ER folding capacity, and by timely removing misfolded proteins from the ER [1], [2], [14], [15]. The elimination of misfolded ER proteins is achieved via the ERAD pathway (also named retrotranslocation). In this process, ER chaperones recognize terminally misfolded proteins and target them to sites in the ER membrane where they are subsequently transferred across the membrane to enter the cytosol. Ubiquitin E3 ligases associated with the ER membrane then catalyze the polymerization of ubiquitin chains on substrates [16]. This allows substrates to be extracted from the ER membrane by a cytosolic AAA ATPase named p97/VCP, which, together with the associated cofactors, shuttles the substrates to the 26S proteasome for degradation [17], [18].

The diverse misfolding signals present in ERAD substrates necessitate the involvement of multiple mechanisms during the initiate stage of retrotranslocation. Indeed, many ER chaperones have been implicated in substrate recognition for distinct classes of misfolded proteins, and several retrotranslocation routes have been proposed to mediate the transfer of different substrates across the ER membrane [17], [18], [19]. Along the same line, a handful of E3 ligases each serve a cohort of client substrates to decorate them with polyubiquitin chains [20], [21]. However, in sharp contrast to the mechanistic diversity in the upstream steps of ERAD, the downstream events appear highly unified as almost all ERAD substrates tested to date use the p97 ATPase for membrane extraction and the proteasome for degradation [22], [23]. Accordingly, inhibition of p97 or the proteasome usually has a more pronounced effect on ER homeostasis than interference with molecules acting in upstream steps.

Given the critical role of ERAD in regulating ER homeostasis, it is conceivable that defects in this process can have significant impact on cell viability, particularly for cells bearing a heavy secretory burden. Accordingly, the ERAD pathway has emerged as a potential target for pharmacological intervention with certain types of tumors. For example, the proteasome inhibitor bortezomib (Velcade™) has been approved for clinical treatment of multiple myeloma and Mantle cell lymphoma (MCL) [24]. The anti-cancer activity of bortezomib can be, at least in part, attributed to ER stress induction as a result of its inhibitory role on ERAD [25], [26], [27], [28], [29], [30]. Moreover, we recently reported that the ERAD specific inhibitor Eeyarestatin I (EerI) can induces cell death in hematologic cancer cells via a mechanism similar to that of bortezomib [31], [32]. Specifically, both EerI and bortezomib induce ER stress, which activates the expression of several CREB/ATF transcription factors including ATF4 and ATF3. EerI and bortezomib also cause the accumulation of polyubiquitinated proteins in cells, leading to a compensatory loss of mono-ubiquitinated histone H2A, an epigenetic mark for transcription repression. ATF4 and ATF3 cooperate with this epigenetic derepression mechanism to upregulate the expression of NOXA, a BH3 domain-containing proapoptotic protein [32]. In this study, we dissect the molecular mechanism underlying the biological action of EerI. Our results indicate that EerI is a bi-modular compound that comprises of two functionally independent domains. An aromatic module in EerI targets it to membranes, allowing a nitrofuran-containing (NFC) module to directly bind to p97 and to interfere with its ER-associated functions. As a result, EerI is a much more specific disruptor of ER homeostasis compared to a compound that only has the NFC domain. These findings elucidate the mechanism by which EerI acts to inhibit ERAD and to induce cell death, and reveal a potential approach to improve drug specificity for cancer therapy that targets ER homeostasis.

## Results

### EerI interacts directly with p97

**Figure 1:**
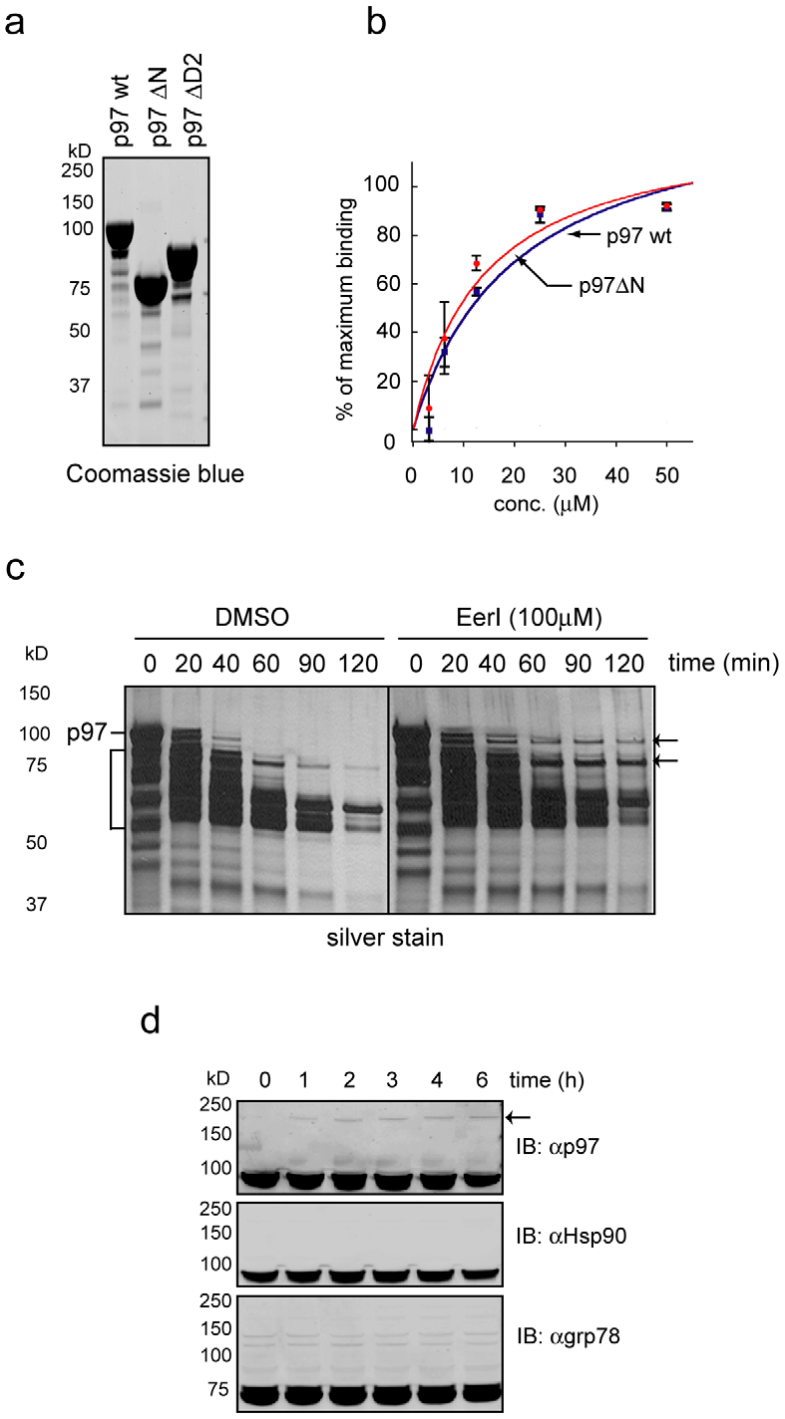
EerI targets the p97 ATPase. **a**, Purified wild type and mutant p97 proteins used in the binding experiments. **b**, Surface plasmon resonance analyses of p97-EerI interaction. Where indicated, a mutant p97 lacking its N-terminal domain (p97 ΔN) was also tested. The binding signals relative to the calculated Rmax from three independent experiments were plotted. Error bar, SD (n = 3). **c**, EerI alters p97 conformation. p97 was incubated with trypsin in the presence or absence of EerI. The digested samples were analyzed by SDS-PAGE and silver staining. Arrows indicate p97 fragments partially protected by EerI. The bracket indicates p97 degradation products. **d**, Whole cell extracts from cells treated with EerI for the indicated time periods were analyzed by immunoblotting with the indicated antibodies. The arrow indicates a species of slow migrating p97 caused by EerI treatment.

EerI was previously found in association with a p97-containing protein complex in EerI-treated cell extract [31], but whether EerI can directly target p97 was unclear. We therefore tested whether EerI could bind recombinant p97 purified from *E coli.* using surface plasma resonance (SPR). When EerI was injected into a CM5 chip immobilized with p97, it generated a concentration dependent response (Figure S1). Plotting the measured response relatively to the calculated maximum response (Rmax) gave rise to an estimated Kd of 5–10 µM (Figure 1a, b). To understand which domain in p97 was critical for EerI interaction, we tested the binding of EerI with two p97 mutants that lacked either the N-terminal co-factor binding domain (p97ΔN) or the second ATPase domain (p97ΔD2). The results showed that EerI had similar affinity to wild type and mutant p97 proteins (Figure 1a, b, data not shown). We concluded from these results that EerI could directly bind p97 most likely at a site in the D1 ATPase domain.

To understand how EerI binding might affect p97 function, we measured the in vitro ATPase activity of p97 in the presence of EerI, but found that EerI had no effect on the p97 ATPase activity (data not shown). We next tested whether binding of EerI to p97 could alter its conformation, which usually resulted in a change in sensitivity to protease digestion. This approach has been used to demonstrate an ATP-induced conformational change in p97 [33]. Interestingly, pre-incubation of EerI with purified p97 caused a delay in the proteolysis of two p97 fragments by trypsin (Figure 1c). This result confirms that EerI can directly interact with p97, and suggests that a conformational change might occur to p97 upon binding to EerI. We also found that in EerI-treated cells a fraction of p97 displayed reduced motility on SDS-PAGE gel. This effect was specific as no such high molecular weight species could be detected for several other ATPases in EerI-treated cells (Figure 1d). Together, our results indicate that EerI may alter p97 conformation to form non-functional p97 oligomers in cells (with a small fraction of such oligomers being resistant to SDS treatment).

### Structure-activity relationship analysis of EerI

**Figure 2:**
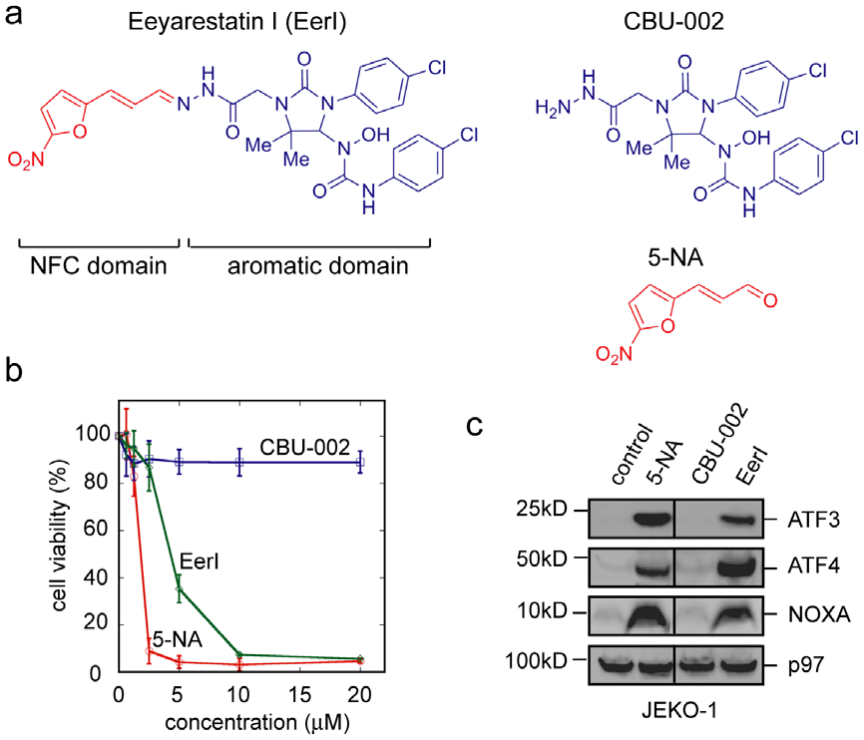
Structure activity relationship (SAR) analysis of EerI. **a**, EerI can be divided into a nitrofuran-containing (NFC) and an aromatic domain, which is represented by compounds CBU-002 and 5-NA (5-nitrofuryl-acrolein), respectively. **b**, Cytotoxic activity of EerI, 5-NA and CBU-002 in JEKO-1 cells as determined by a MTT assay. Error bar, SD (n = 3). **c**, The induction of ER stress and NOXA expression requires the NFC domain of EerI. Whole cell extracts from JEKO-1 cells exposed to the indicated agents (10 µM for EerI, CBU002, and 2.5 µM for 5-NA) were subjected to immunoblotting analyses.

To further understand the chemical basis of EerI action, we divided EerI into two structural subunits defined as a nitrofuran-containing (NFC) moiety and an aromatic domain, respectively (Figure 2a). We compared the cytotoxicity of EerI to that of 5-NA (5-nitrofuryl-acrolein) and CBU-002, which corresponded to these two subunits. JEKO-1 cells, a MCL-derived cancer cell line, were treated with these compounds and cell viability was measured by a colorimetric assay using MTT (3-(4,5-Dimethylthiazol-2-yl)-2,5-diphenyltetrazolium bromide, a tetrazole). EerI induced cell death in JEKO-1 cells with an IC50 of 4±1.2 µM, as demonstrated previously [32]. 5-NA was also toxic with a moderately reduced IC50 (∼1 µM), whereas CBU-002 was completely inactive (Figure 2b). We next tested whether 5-NA and CBU-002 could influence the expression of ATF3, ATF4 and NOXA, as has been demonstrated for EerI [32]. Immunoblotting experiments revealed that like EerI, 5-NA upregulated the expression of ATF3, ATF4, and NOXA. By contrast, CBU-002 lacked this activity (Figure 2c, Figure S2). We also tested which of these domains were essential for ERAD inhibition by monitoring the degradation of MHC class I heavy chain in US11-expressing cells treated with these compounds. US11 is a human cytomegalovirus-encoded protein that co-opts the ERAD pathway to degrade newly synthesized MHC class I heavy chain at the ER membrane [34]. Pulse chase experiments showed that cells treated with 5-NA exhibited a significant delay in the turnover of MHC class I heavy chain similarly to cells treated with EerI, whereas CBU-002 had no effect (Figure S3). Together, these results strongly suggest that the NFC domain is the functional group responsible for the ERAD inhibition and cytotoxic activities of EerI.

### The NFC domain of EerI can interact with p97

**Figure 3:**
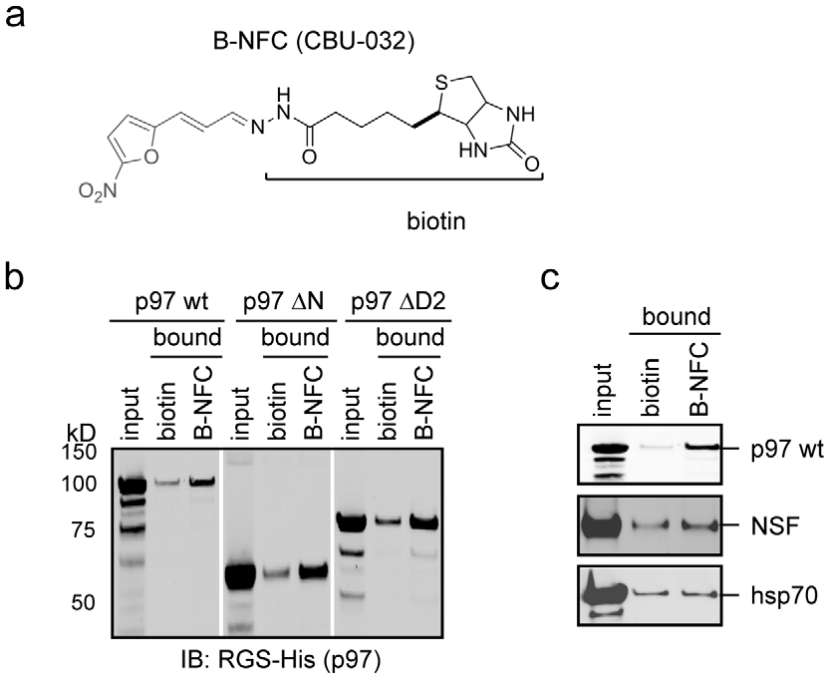
EerI interacts with p97 via the NFC domain. **a**, The structure of biotinylated NFC (B-NFC). **b**, Monomeric avidin beads immobilized with CBU-032 or biotin were incubated with the indicated recombinant proteins. The bound materials were eluted with biotin and analyzed by SDS-PAGE and immunoblotting. **c**, As in **b**, except that the indicated His-tagged proteins were tested.

We hypothesized that EerI might bind p97 via the NFC domain, which would explain why these two compounds have similar activities in cells. The molecular weight of 5-NA is too small for SPR analysis. We therefore conjugated a biotin moiety to 5-NA to generate a biotinylated NFC domain (B-NFC) (Figure 3a). This allowed us to immobilize the NFC domain on avidin-conjugated beads and test its binding to purified p97 by co-precipitation. We used biotin as a negative control, which under our assay condition precipitated a small amount of p97, presumably due to non-specific interaction. B-NFC, however, consistently precipitated more p97 (Figure 3b). Consistent with our SPR studies on EerI, p97 mutants that lacked either the N-terminal domain (p97ΔN) or the D2 ATPase domain (p97ΔD2) also bound to 5-NA (Figure 3b). This interaction was specific to p97 as a homologous AAA ATPase termed NSF (N-ethylmaleimide-sensitive factor) did not show significant binding to B-NFC, neither did the heat shock protein Hsp70 (Figure 3c). These results demonstrate that EerI can directly interact with p97 via the NFC domain.

### Target specificity of EerI in cells

**Figure 4:**
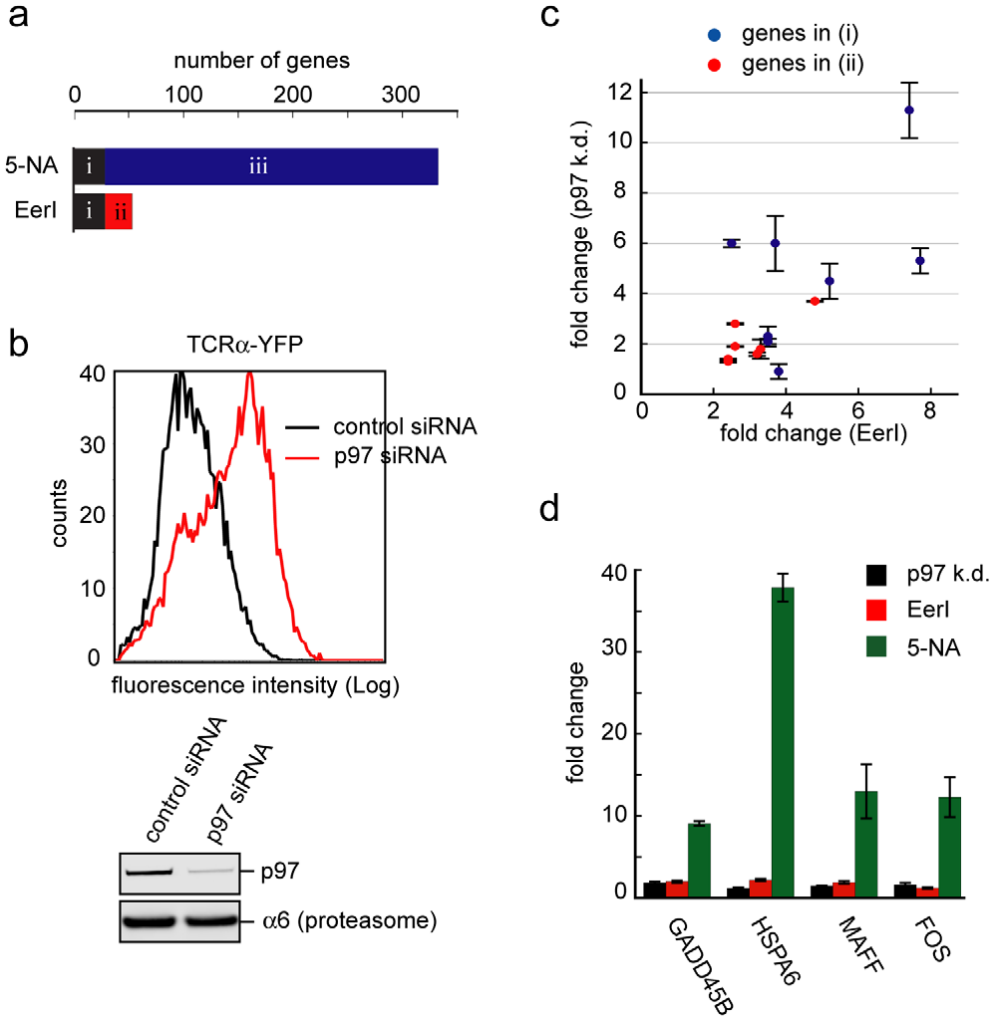
p97 is a target of both EerI and 5-NA in cells. **a**, 5-NA has a broader effect on gene expression than EerI. The number of genes up- or down-regulated by at least 2-fold upon treatment with EerI (10 µM, 10 h) or 5-NA (10 µM, 10 h) in 293T cells was plotted. These genes can be divided into three categories including 29 genes affected by both EerI and 5-NA (i), 25 genes affected only by EerI (ii) and 306 genes affected only by 5-NA (iii). **b**, Knock-down of p97 inhibits the degradation of TCRα-YFP as determined by flow cytometry. A fraction of the cells were used to verify the knock-down efficiency by immunoblotting with a p97 antibody. **c**, **d**, The effect of p97 depletion on the expression of EerI and 5-NA signature genes. **c**, The expression of selected genes in the categories i (8 genes, blue dots) and ii (7 genes, red dots) was determined by qRT-PCR using RNA prepared from p97 knock-down (k.d.) and control cells. The fold change upon p97 depletion was plotted against EerI-induced fold change. Error bars, SD (n = 3) **d**, The expression of 4 genes in the category (iii) in p97 knock-down cells, or cells exposed to EerI (10 µM) or 5-NA (10 µM) was determined by qRT-PCR. Untreated cells were used to determine the basal expression of these genes, which was used to calculate the fold change.

To further confirm that both EerI and 5-NA can influence p97 function in cells, we compared the gene expression pattern of EerI-treated cells to that of 5-NA-treated cells using whole genome array hybridization. We presumed if two chemicals target a same gene, the change in gene expression profile in response to these compounds should overlap significantly and genes affected by these compounds should be similarly influenced when the target gene is inhibited by a siRNA-mediated knock down approach. We chose 293T cells for this study because 293T cells are highly transfectable, making them well suited for genetic manipulation by siRNA. 5-NA treatment dramatically altered the gene expression landscape, resulting in changes in the expression of a large number of genes. Interestingly, the number of genes affected by EerI was significantly smaller (Figure 4a; Table S1). As expected, a large number of genes affected by EerI were similarly affected by 5-NA. We defined these genes as cohort (i). Pathway analyses showed that UPR-associated genes were enriched in this cohort. By contrast, genes only affected by EerI (cohort ii) or 5-NA (cohort iii) did not display the signature of UPR activation. Instead, they represented other pathways unrelated to ER homeostasis (Figure S4; unpublished data). These results suggested that 5-NA has a broader impact on cell physiology than EerI, suggesting that it may have additional targets other than p97. The conjugation of the aromatic module to 5-NA seems to restrict its action, making ER a primary target of the resulting compound EerI.

To test whether the gene expression signature shared by 5-NA and EerI was indeed a result of p97 inhibition, we knocked down p97 in 293T cells. The knock-down efficiency was confirmed both by immunoblotting with an anti-p97 antibody and by monitoring the stabilization of the model ERAD substrate TCRα in p97 depleted cells (Figure 4b). We analyzed the effect of p97 depletion on the expression of representative genes upregulated by EerI and 5-NA including 8 genes in cohort (i), 7 genes in cohort (ii), and 4 genes in cohort (iii). qRT-PCR analyses showed that all genes except for one in cohort (i) were significantly induced upon p97 depletion (>twofold). By contrast, only 2 out of the 7 genes in cohort (ii) were significantly upregulated in p97 knock-down cells (Figure 4c), and none of the genes tested in cohort (iii) were induced by knock-down of p97 (Figure 4d). These results confirm that p97 is a target of both EerI and 5-NA. Because the expression of more than 50% of EerI signature genes (cohort i) are similarly affected by p97 depletion, p97 appears to be a major target of EerI. By contrast, the majority of the 5-NA-affected genes (cohort iii) do not seem to result from p97 inhibition. Thus, 5-NA must have additional targets other than p97 in cells.

### The aromatic module recruits EerI to the ER membrane

**Figure 5:**
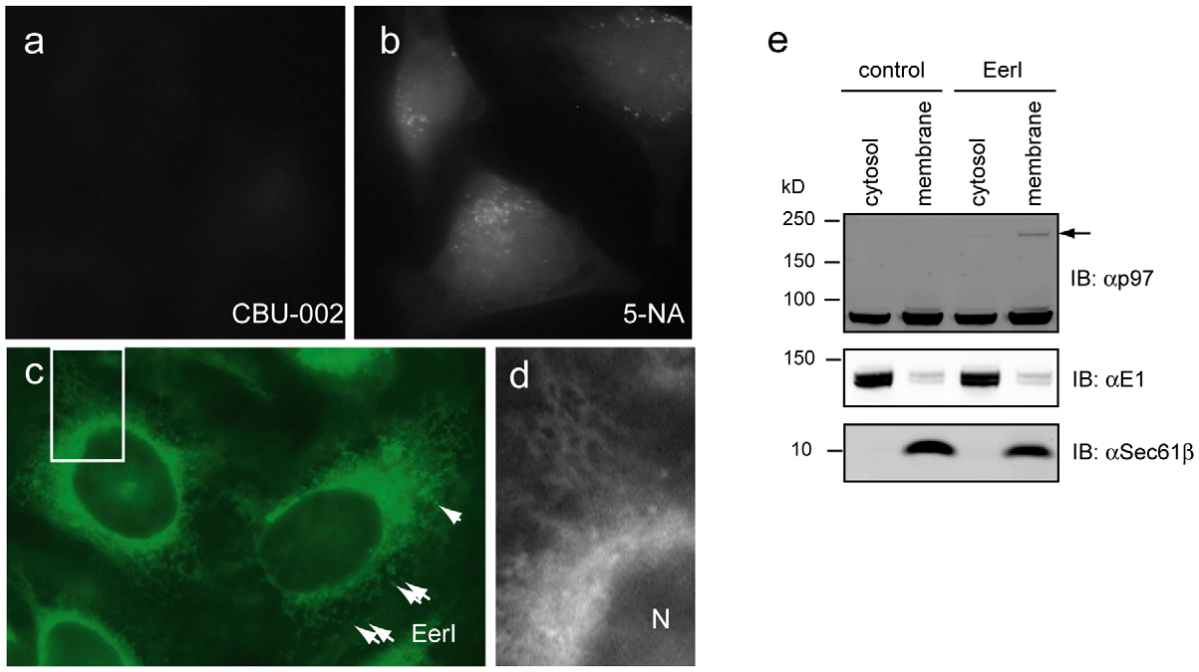
EerI is associated with the ER membrane. **a–c**, HeLa cells were treated with the indicated compound (10 µM, 1 h) and imaged with a fluorescence microscope. Arrows in **c** indicate some EerI-stained vesicles that may be derived from the ER or the endocytic system. **d**, An enlarged view of the indicated area in **c** showed the localization of EerI to a perinuclear reticulum-like membrane compartment in cells. N, nucleus. **e**, p97 preferentially affects membrane associated p97. 293T cells were either untreated or treated with EerI (10 µM 6 h). Cells were fractionated into membrane and cytosol fractions. Proteins extracted from these fractions were analyzed by immunoblotting with the indicated antibodies. Note that the slow migrating p97 species (indicated by the arrow) induced by EerI is primarily in the membrane fraction.

To understand how the aromatic domain improves EerI target specificity, we examined the subcellular localization of EerI and 5-NA, taking advantage of their intrinsic fluorescence properties [31]. CBU-002 was used as a negative control because it was not fluorescent in vitro and failed to generate a fluorescence signal in cells (Figure 5a). 5-NA-treated cells showed a uniform staining pattern with some punctae (Figure 5b), suggesting that 5-NA was ubiquitously distributed throughout a cell. By contrast, EerI-treated cells displayed a strong fluorescence signal in a perinuclear reticulum-like pattern, indicative of ER localization (Figure 5c, d). EerI also labeled some vesicles in a perinuclear region that might be derived from the endocytic system (Figure 5c). Consistent with the localization study, we found that in EerI-treated cells, the SDS-resistant p97 oligomer species was detectable only in the membrane fraction but not in the cytosol fraction (Figure 5e). Together, these results suggest that EerI can associate with the ER membrane to selectively interfere with the function of membrane-associated p97.

**Figure 6:**
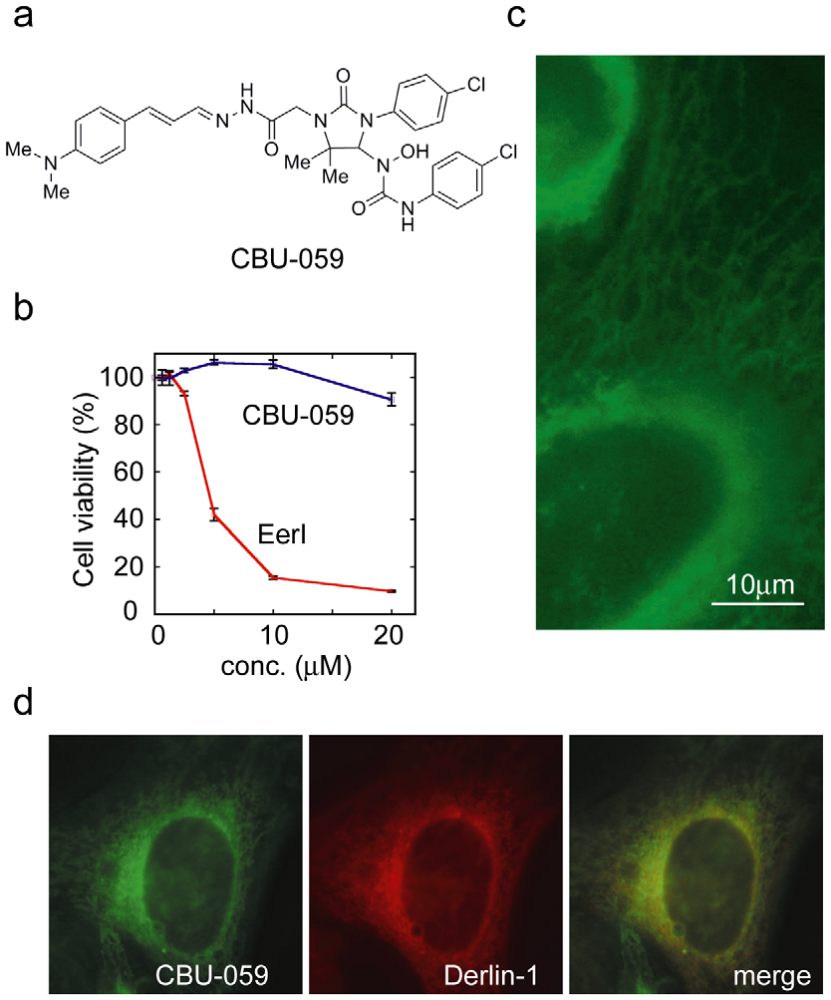
The aromatic domain targets EerI to the ER membrane. **a**, The structure of CBU-059. **b**, cytotoxicity of CBU-059. **c**, CBU-059 stains a perinuclear reticulum-like membrane compartment. HeLa cells stained with CBU-029 (3 µM, 1 h) were imaged by a fluorescence microscope using a FITC filter set. **d**, CBU-029 co-localizes with the ER membrane protein Derlin-1.

To further characterize the membrane association of the aromatic domain of EerI, we replaced the NFC domain with several aldehydes that might give rise to a stronger fluorescence signal. We predicted that the resulting compounds were likely to be non-toxic as they did not contain the NFC moiety, but they should be localized to the ER if the aromatic domain of EerI was capable of binding to the ER membrane. One of the resulting compounds CBU-059 (Figure 6a) indeed produced a stronger fluorescence signal than EerI and had little effect on the viability of JEKO-1 cells (Figure 6b). When exposed to CBU-059, cells displayed perinuclear ER-like staining pattern resembling EerI-treated cells (Figure 6c). Double labeling experiment showed that CBU-059 co-localized with Derlin-1 (Figure 6d), an ER resident protein [35], [36]. These results demonstrate that the aromatic domain in EerI is sufficient to recruit EerI to the ER membrane.

### Identification of a new bifunctional ERAD inhibitor

**Figure 7:**
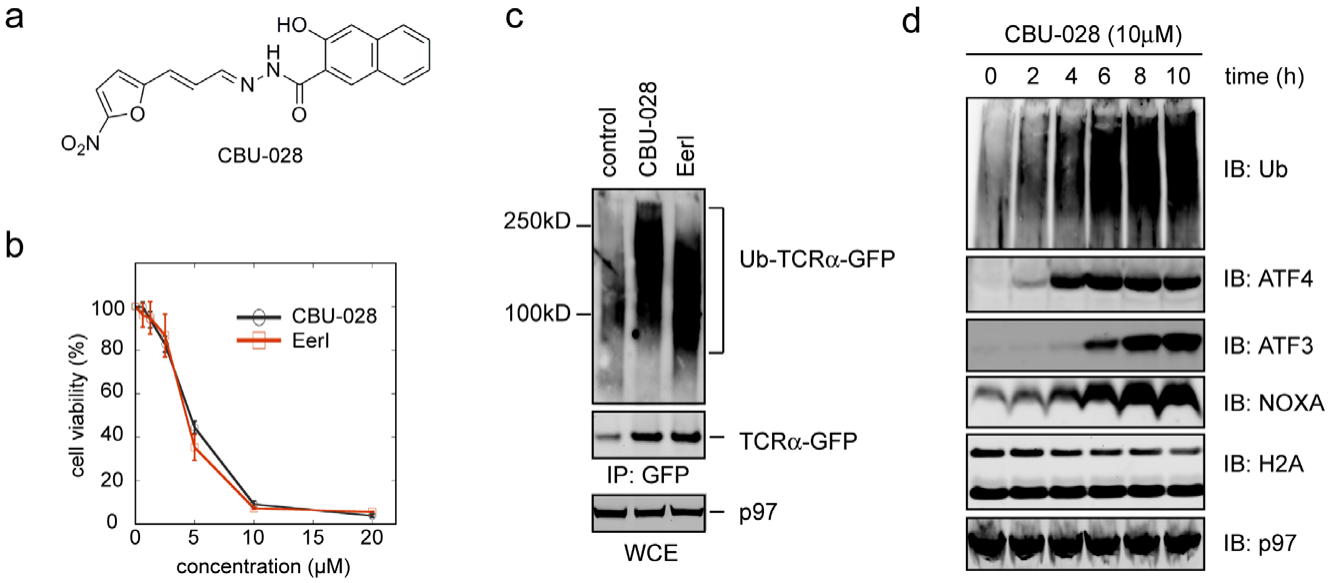
CBU-028 has biologic activities resembling EerI. **a**, Structure of CBU-028. **b**, CBU-028 has a similar cytotoxic activity as EerI in JEKO-1 cells. Cells were treated with the indicated compounds and cell viability was measured by a MTT assay. Error bar, SD (n = 3). **c**, CBU-028 stabilizes the ERAD substrate TCRα-YFP in polyubiquitinated forms. 293T cells stably expressing TCRα-YFP were treated with the indicated compounds (10 µM) or as a control with DMSO for 10 h. TCRα-YFP was immunoprecipitated from the cell extract. The precipitated material was analyzed by SDS-PAGE and immunoblotting. **d**, CBU-028 has similar biological activities as EerI. JEKO-1 cells were treated with 10 µM CBU-028 for the indicated time periods. Whole cell extract was analyzed by immunoblotting with antibodies against the indicated proteins.

The bi-modular nature of EerI predicts that it should be possible to replace the aromatic domain of EerI with a structurally simpler membrane-targeting group, which should give rise to a compound with similar activity to EerI. Developing a simpler compound with similar activity as EerI is a critical issue because of the difficulty in synthesizing EerI in large scale. We therefore generated a chemical library that carried the same NFC domain but with varied benzene-containing groups. We used the cytotoxicity assay as a first screen, which demonstrated that a large number of these compounds remained toxic to cells (Table S2), consistent with the idea that the NFC domain was responsible for the cytotoxicity. We chose one of these chemicals designated as CBU-028 for further characterization because its cytotoxicity in JEKO-1 cells was identical to that of EerI (Figure 7a, b).

We first tested whether CBU-028 could have an inhibitory effect on ERAD using the model substrate TCRα. Cells stably expressing YFP-tagged TCRα were treated with EerI and CBU-028. The substrate TCRα-YFP was immunoprecipitated from cell extracts under denaturing condition. Immunoblotting showed that EerI and CBU-028 stabilized TCRα to a similar degree. Importantly, a significant fraction of TCRα in CBU-028-treated cells carried polyubiquitin conjugates just like in EerI-treated cells (Figure 7c). The ubiquitin chains on TCRα from CBU-028-treated cells appeared to be longer than that in EerI-treated cells, which might be due to small differences in the inhibitory kinetics or unknown off-target effect of these compounds (see below). Thus, CBU-028 can inhibit ER-associated protein degradation at a step similar to EerI. Importantly, CBU-028 also upregulated the expression of the ER stress-associated transcription factors ATF3 and ATF4, caused accumulation of polyubiquitinated proteins and downregulation of ubiquitinated histone H2A (Figure 7d). These changes were previously reported in EerI-treated cells, suggesting that CBU-028 has biological activities similar to EerI. Accordingly, CBU-028 also activated NOXA expression (Figure 7d), and a JEKO-1 derived cell line stably expressing a NOXA shRNA was more resistant to CBU-028-induced cell death (data not shown).

### Target specificity and localization of CBU-028

**Figure 8:**
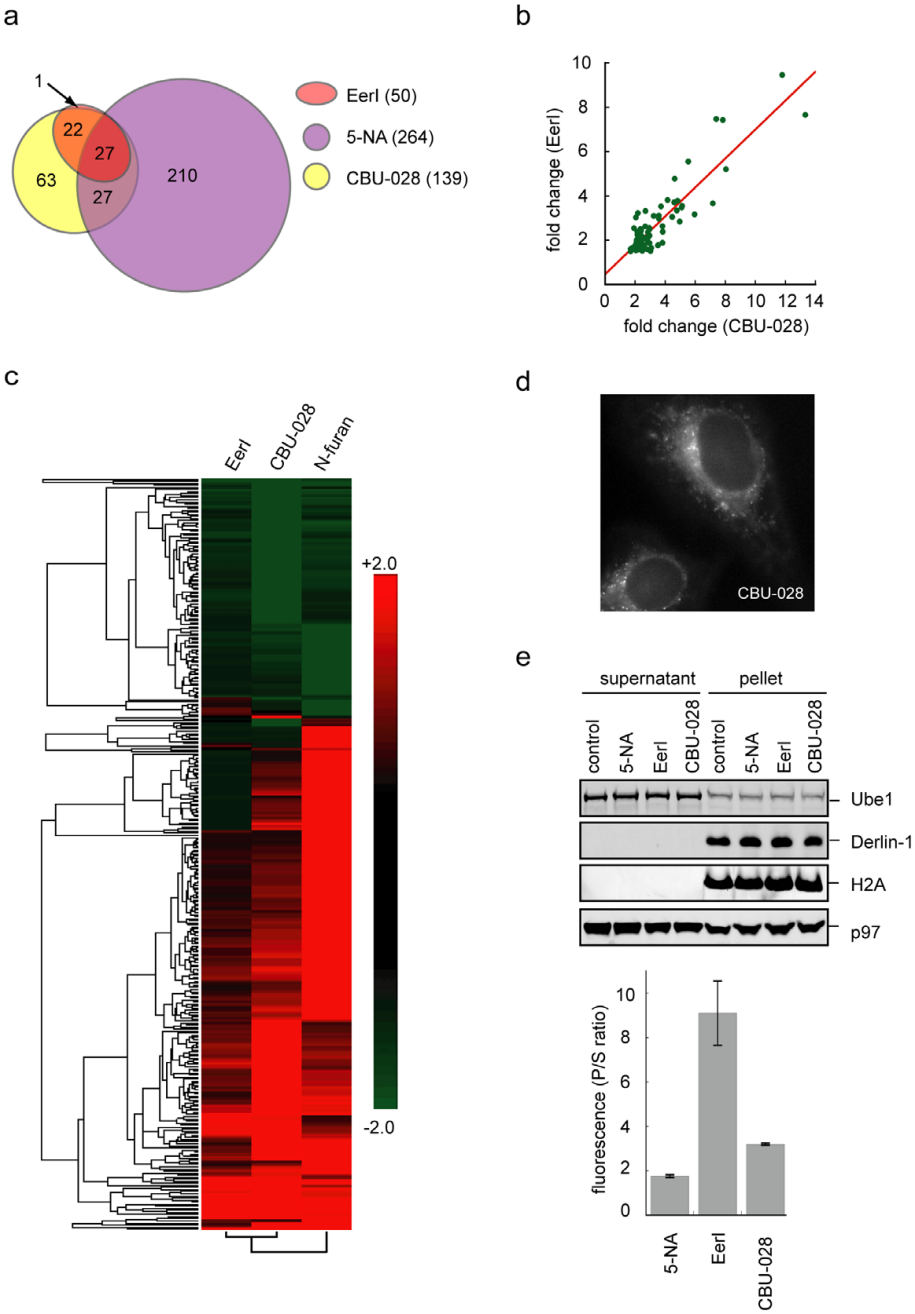
Target specificity and subcellular distribution of CBU-028. **a**, Microarray analyses of gene expression profiles of EerI-, 5-NA-, and CBU-028-treated 293T cells. Shown is a Venn diagram indicating the numbers of genes significantly induced (>2 fold, p value <0.05) by these compounds. **b**, EerI-activated genes are also similarly induced by CBU-028. The fold change for EerI-induced genes was plotted against the fold change induced by CBU-028. **c**, A heat map representation of genes whose expression is affected by EerI, 5-NA, or CBU-028. Both significantly up- or down-regulated genes (>2 fold, p value <0.05) are included. Note that clustering analysis indicates that CBU-028 resemble EerI more than 5-NA. **d**, CBU-028 also accumulated at a perinuclear reticulum-like compartment. HeLa cells were treated with 10 µM CBU-028 and imaged by a fluorescence microscope. **e**, Subcellular fractionation analysis shows that CBU-028 is moderately enriched in the membrane. HeLa cells exposed to the indicated compounds were permeabilized and then fractionated into a supernatant fraction containing the cytosol and a pellet fraction comprising the ER membrane and the nucleus. The distribution of the indicated proteins in these fractions was determined by immunoblotting. The fluorescence signal in the fractions was measured by a fluorimeter. The signal in the corresponding fractions from untreated control cells was taken as the background. Shown is the ratio between the fluorescence signal in the pellet fraction and that in the cytosol. Error bars represent the average of two independent experiments.

To define the target specificity of CBU-028, we performed the whole genome expression microarray analysis for CBU-028-treated 293T cells and compared the CBU-028's effect on gene expression to that of EerI or 5-NA. These analyses revealed that CBU-028 had smaller impact on gene expression than 5-NA, although it still affected more genes than EerI (Figure 8a; Table S1). A close examination of genes whose expression was significantly altered by these compounds revealed that CBU-028 was more similar to EerI than 5-NA (Figure 8a–c). Taking the upregulated genes as an example, all but one gene activated by EerI was also induced by CBU-028 (Figure 8a), and the fold induction by these two compounds correlated precisely with each other (Figure 8b). By contrast, 61% of the CBU-028-induced genes were not affected by 5-NA, and an even larger percentage of 5-NA upregulated-genes displayed no significant change in cells treated with CBU-028 (Figure 8a). Clustering analysis of significant genes (including both up- and down-regulated genes, fold change ≥2.0; p value ≤0.05) further confirmed that CBU-028 was more closely related to EerI than to 5-NA (Figure 8c). Together, these results suggested that CBU-028 has improved target specificity when compared to 5-NA. As a result, it has activities more closely resembling EerI.

We next examined the subcellular distribution of CBU-028. Like EerI, CBU-028 was also autofluorescent, but the fluorescence intensity was ∼5fold less than EerI (unpublished data). Nonetheless, cells treated with CBU-028 displayed detectable fluorescence signals in a perinuclear reticulum-like pattern resembling that in EerI-treated cells (Figure 8d). To further confirm the association of CBU-028 and EerI in membranes, we treated cells with these compounds or 5-NA and then permeabilized cells using a low concentration of the detergent digitonin. Cells were then fractionated into a soluble fraction containing the cytosol and a pellet fraction containing the ER membrane and nuclei. The fluorescence signal in these fractions was measured and the ratio between the signal in the pellet and that in supernatant fractions was calculated. Consistent with the imaging experiments, the fluorescence signal of 5-NA was not significantly concentrated in the membrane pellet fraction (Figure 8e). The ∼1.8fold enrichment in the pellet fraction for 5-NA was likely caused by the nucleus-localized 5-NA. By contrast, the EerI fluorescence signal was dramatically enriched in the pellet fraction (>9fold enrichment). CBU-028 also showed ∼3.2fold enrichment in the membrane fraction. Although the membrane enrichment of CBU-028 was less significant than EerI, our gene expression analyses and activity studies suggest that the moderate enrichment of CBU-028 to the ER membrane is sufficient to allow CBU-028 to acquire similar activities as EerI and to obtain improved target specificity.

## Discussion

### EerI is a bifunctional inhibitor that has an ER targeting domain

EerI is the only known chemical inhibitor of the ERAD pathway [31], [37], but the mechanism of its action was unclear. In this study, we demonstrate that EerI is comprised of two functional domains, a nitrofuran-containing (NFC) domain and an aromatic domain. The NFC domain of EerI is essential for its ERAD inhibitory and cytotoxic activities. The aromatic domain of EerI is non-toxic by itself, but it can recruit EerI to the ER membrane. Since the aromatic domain of EerI can be replaced with a structurally distinct membrane-targeting group and the resulting compound retains almost all the activities of EerI, we assume that the major contribution of this domain is to localize EerI to the ER membrane. The functions of the two EerI subdomains can be uncoupled as they can be individually manipulated without affecting the activity of the other. For example, the NFC domain remains cytotoxic when conjugated to diverse hydrazines. Conversely, replacement of the NFC domain in EerI with another aldehyde can abolish its cytotoxic activity without changing the ER localization. These results reveal a novel chemical structural element that can be used to target small molecule inhibitors to the ER membrane. Given that ER homeostasis has emerged as an important therapeutic target for cancer treatment, our findings may aid the development of new anti-cancer drugs that target ER-associated functions. For example, several ER membrane-anchored signaling molecules including Ire1 and PERK are now being evaluated as potential anti-cancer targets [8]. Bi-modular compounds comprising an inhibitory “warhead” that targets these signaling molecules and an ER-localizing moiety such as the one in EerI may be superior to non-localized inhibitors as the former may have less undesired off-target effects (see below). Our study also shows that when conjugated to a non-toxic fluorescent unit, the aromatic domain of EerI can be used as a fluorescent probe to label the ER with little effect on cell growth. In this regard, CBU-059 and compounds of its class may be well suited for studying ER morphology and functions in live cells.

### EerI interacts with p97 via the NFC domain

We identify the p97 ATPase as a target of EerI and show that the nitrofuran domain in EerI is required for interaction with p97. The binding appears to occur to the D1 domain of p97. It is noteworthy that the AAA ATPase domain is highly conserved among this ATPase family. Nonetheless, the NFC domain of EerI appears to be able to discriminate between p97 and a closely related protein NSF. This suggests that EerI is unlikely to substantially affect the functions of other AAA proteins in cells. A recent study suggested that EerI may also target the Sec61 complex to inhibit protein translocation into the ER [38]. The reported inhibition of protein translocation in vitro requires a high concentration of EerI (IC50>70 µM). At such a high concentration, the drug may have non-specific interactions. Inhibition of ER protein expression was observed in intact cells treated with 5–10 µM EerI [38]. This might be due to reduced protein translocation or translation as a consequence of ER stress induction, as demonstrated previously [14], [39]. Alternatively, the enrichment of EerI, a compound with a hydrophobic membrane binding moiety, in the ER membrane may alter the fluidity of the membrane to indirectly affect protein import into the ER. Indeed, it has been demonstrated that loading the ER with cholesterol inhibits protein translocation [40]. Future studies will be required to clarify whether or not EerI can also directly target the Sec61 complex.

### Membrane localization and target specificity

Nitrofurans define a class of compounds with the signature component of a furan ring and a nitro group. The NFC domain in EerI corresponds to 5-nitrofuryl-acrolein (5-NA), which is an unsaturated aldehyde carrying a nitrofuran group. Early studies demonstrated that 5-NA and its structurally related compounds are highly mutagenic [41], [42]. We demonstrate here that the NFC domain in EerI is capable of interacting with p97, which explains the observed effect of 5-NA on ERAD. However, consistent with early toxicity studies, the effect of 5-NA on cells appears to be pleiotropic as it also affects many pathways unrelated to ER homeostasis. Gene expression studies show that most genes activated by 5-NA are not p97 signature genes, suggesting that it can target other proteins in addition to p97. Interestingly, the addition of the aromatic domain to 5-NA alters its target specificity, making the resulting compound EerI a more specific inhibitor for ER homeostasis. Compared with 5-NA, EerI only influences the expression of a small set of genes, most of which result from ER stress induction. Our qRT-PCR analyses estimate that at least 50% of genes affected by EerI are p97 signature genes, suggesting that p97 is a primary target of EerI. The localization of EerI to the ER membrane by the aromatic domain appears to be responsible for the improved target specificity because replacing this domain with another benzene-containing moiety that has a moderate affinity for the ER membrane produces a compound that has activities closely resembling EerI. The localization of EerI to the ER seems to limit its action to only membrane-bound p97. This can explain why ERAD inhibition and ER stress are the two major phenotypes of cells exposed to EerI even though p97 is abundant in the cytosol and have functions unrelated to the ERAD [23], [43], [44], [45]. The aromatic domain of EerI may also improve targeting specificity by restricting EerI from interacting with cellular targets of 5-NA either as a result of its membrane association or steric hindrance.

In summary, our studies uncover a class of bifunctional compounds that associate with the ER membrane to disrupt ER homeostasis. We propose that localizing compounds to the ER membrane may be an effective strategy to improve the specificity for cancer therapeutics that targets ER homeostasis.

## Materials and Methods

### Proteins, antibodies, and chemicals

Wild type p97 and mutant proteins were purified as previously described [46]. Hsp70 protein was purchased from Assay Designs, Inc. (Ann Arbor, MI). Immunoblotting experiments were carried out using the following antibodies: FLAG antibody (Sigma), NOXA antibody (EMD), ATF3 (Santa Cruz), histone H2A (Abcam). The ATF4 antibody was described previously [32]. EerI was synthesized by the NIDDK Chemical Biology Core facility. 5-NA was purchased from TCI America. The synthetic protocols for the hydrazone library and EerI will be reported elsewhere. EerI can also be purchased from Chembridge Corporation (San Diego, CA) or Tocris Bioscience (Ellisville, MO). Control siRNA and the Smart pool siRNA targeting p97 were purchased from Dharmacon (Lafayette, CO).

### Mammalian cell experiments

HeLa and 293T cells from ATCC were maintained according to the standard procedures. JEKO-1 cells were kept in RPMI, 10% FCS as previously described. NOXA and control stable knock-down JEKO-1 cells were described previously [32]. PBMC from normal volunteers were obtained from the Department of Transfusion Medicine, NIH and PBMC from CLL patients were obtained under NIH protocol 97-C-0178 with informed consent. Transfection was done with lipofectamine 2000 (Invitrogen) for 293T accordingly to manufacture's instruction. Pulse chase and denaturing immunoprecipitation were carried out as described previously [47], [48]. Subcellular fractionation experiments were carried out as previously described [49]. Briefly, HeLa cells were harvested and washed with phosphate-buffered saline. Cells were then treated on ice with the buffer PB (25 mM Hepes, pH 7.2, 115 mM potassium acetate, 5 mM sodium acetate, 2.5 mM magnesium acetate, 0.5 mM EGTA, and a protease inhibitor cocktail) that also contain 0.028% digitonin. Cells were centrifuged at 20,000×g for 10 min. The cytosolic supernatant fraction was removed and the nucleus and membrane pellet fraction was washed twice with the PB buffer. The pellet fraction was finally resuspended in the PB buffer. The fluorescence intensity in these fractions was measured by the Victor 3 plate reader using the Ex482 and Em523 filter set. Cell viability assay were performed using the MTT (3-(4,5-dimethylthiazol-2-yl)- 2,5-diphenyl tetrasodium bromide) reagent (Chemicon, Temecula, CA) as described previously [50]. Briefly, JEKO-1 cells were incubated with the drugs at the indicated concentration for 48 h. MTT was added to the medium at the concentration of 0.5 mg/ml. After 4 h incubation, the sample absorbance at 570 nM and 650 nM were measured using a Victor 3 plate reader (Perkin Elmer, Waltham, MA) and ratio of drug-treated samples vs. the untreated control was used to determine cell viability. Immunoblotting experiments were performed according to standard protocol. Fluorescence labeled secondary antibodies (Invitrogen, Carlsbad, CA) were used for detection. Fluorescent bands were imaged and quantified on a LI-COR Odyssey infrared imager using the software provided by the manufacture. Fluorescence images were obtained using a Zeiss Axiovert200 fluorescence microscope equipped with a 63X oil immersion Plan Apochromat objective and standard filter sets (FITC and rhodamine).

### Co-precipitation experiment

B-NFC was immobilized on monomeric avidin beads by incubating 50 µM compound with the beads in a binding buffer containing 10 mM HEPES, pH 7.2, 150 mM sodium chloride, 0.005% Surfactant P20. The beads were then incubated with the 0.5 µM purified p97 proteins in 500 µl binding buffer at 4 degree for 1 h. The beads were washed for 3 times with the binding buffer and bound materials were eluated by incubating the beads with binding buffer comprising 4 mM biotin.

### RNA preparation, array hybridization and qRT-PCR

Cells were treated with EerI, CBU-028, or 5-NA each at 10 µM in duplicates for 10 h. Total RNA was extracted using TRIzol reagent (Invitrogen, Carlsbad, CA), and subsequently purified using an RNeasy MinElute Cleanup kit (QIAGEN, Valencia, CA). For array hybridization, RNA samples were processed and analyzed by the NIDDK Microarray Core Facility. Affymetrix gene expression analysis array for the human HG-U133A_2_0 were used (Affymetrix). The microarray signals were analyzed using the Affymetrix RMA algorithm. Up- and down-regulated genes were selected based on P values of <0.05 and fold change >2.0 or <−2.0 as assessed by ANOVA with Partek Pro software (Partek). Genes that were upregulated by >1.5 were sometimes used in analysis as indicated in the figure legend. To determine specific pathways, gene pathway analysis were conducted using the Genego program available at http://trials.genego.com/cgi/index.cgi using the statistical significant ANOVA gene list (p<0.05, fold change>1.5) represented on the chip. Microarray data is MIAME compliant and that the raw data has been deposited in the Gene Expression Omnibus (GEO) database (accession no. GSE23849) (http://www.ncbi.nlm.nih.gov/geo/query/acc.cgi?token=xjkpxkuiiyameho&acc=GSE23849).

For quantitative RT-PCR, cDNA was synthesized using the SuperScript™ First-Strand kit (Invitrogen, Carlsbad, CA). Real-time PCR was performed in triplicate using the SYBR green PCR Master mixture. β-actin was used for normalization.

### Surface plasmon resonance (SPR) interaction analysis

Surface plasmon resonance interaction experiments were carried out at 25 degree on a Biacore T100 system (GE healthcare). Recombinant wild type or mutant p97 was covalently attached to a carboxymethyl dextran-coated gold surface (CM5 Chip; GE healthcare) using an amide couple kit following a manufacture suggested protocol. Briefly, the carboxymethyl group of dextran was activated with N-ethyl-N'-(3-dimethylaminopropyl) carbodiimide (EDC) and N-hydroxysuccinimide (NHS). p97 in a buffer containing 10 mM sodium acetate (pH 4.5) was injected at a flow rate of 10 µl/min with 2 min contact time and p97 was immobilized at a level of ∼8,000RU. Any remaining reactive sites in the flow cell were blocked by ethanolamine. The drug association and dissociation were monitored at a flow rate of 20 µL/min with the compound concentration ranging from 3.125 µM to 50 µM. Analysts were prepared in a buffer containing 10 mM HEPES (pH 7.4), 150 mM NaCl, 0,005% v/v Surfactant P20. Regeneration was achieved by extended washing with the analyte buffer. SPR signal was normalized using a reference flow cell containing no p97. For each compound, three independent measurements were made. To calculate the affinity of EerI to p97, we fitted the data from each binding experiment with a Michaelis-Menton model using the KaleidaGraph software. The Rmax was then calculated, and the binding relative to the Rmax from three independent experiments was averaged. The averaged binding data were used to calculate the Kd.

### Limited trypsin digestion experiment

The purified p97 protein (1.5 µg) was incubated at in a buffer containing 200 ng trypsin in 50 µl volume. Samples were taken out at different time points and mixed with the Laemmli buffer before SDS-PAGE and silver staining analyses.

## Supporting Information

Figure S1 **SPR analysis of EerI-p97 interaction.** Representative binding curves show direct binding of EerI to p97. R.U. response unit.

Figure S2 **Induction of ER stress and NOXA expression by EerI and 5-NA.** 293T cells were treated with various compounds at the indicated concentration for 8 h. Whole cell extract was analyzed by immunoblotting with the indicated antibodies.

Figure S3 **5-NA but not CBU-002 inhibits the degradation of MHC class I heavy chain in US11 cells.** US11 cells treated with the indicated compound (10 µM) were pulse labeled in a medium containing 35S-Met/Cyc then incubated in a chase medium containing excess unlabeled Met/Cys. MHC class I heavy chain was immunoprecipitated from cell extract and analyzed by SDS–PAGE and autoradiography.

Figure S4 **Pathway analyses of EerI and 5-NA signature genes.** Genes with fold change ≥1.5 from ANOVA analysis (p value ≤0.05) were used for pathway analyses by the commercial software MetaCore. Shown is a histogram indicating the top 10 cellular processes significantly represented by genes affected by both Eer1 and 5-NA. For comparison, the relative representation of the corresponding pathway by the unique genes for 5-NA or Eer1 was shown in red and in blue, respectively.

Table S1 **A list of genes whose expression is significantly affected by EerI or 5-NA.** (Fold change >1.5, p value <0.05 from a biological replicate).

Table S2 **A list of compounds tested in this study.** JEKO-1 cells were treated with each of the listed compounds at concentrations ranging from 1.25 µM to 20 µM. Cell viability was determined by the MTT assay and used to obtain concentration-response curve. IC50 was extracted by linear interpolation.
