# Supplementary figures and images for: The ERAD Inhibitor Eeyarestatin I Is a Bifunctional Compound with a Membrane-Binding Domain and a p97/VCP Inhibitory Group
Source: PLoS One. 2010 Nov 12;5(11):e15479. doi: 10.1371/journal.pone.0015479 (PMC2993181; doi:10.1371/journal.pone.0015479)

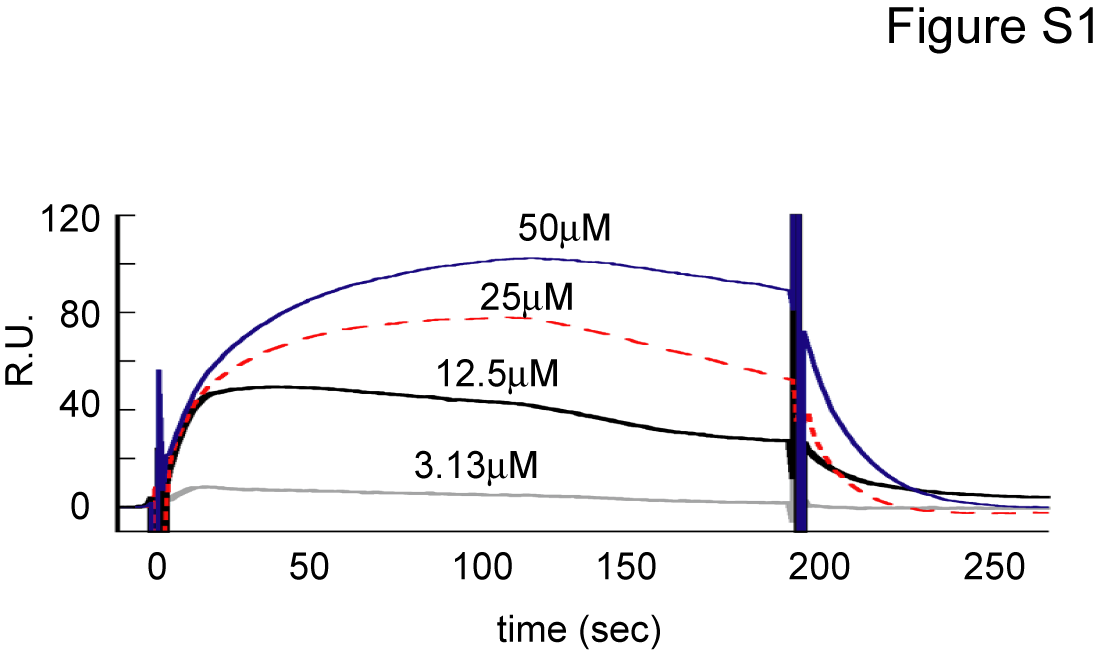

Supplement: Figure S1 — SPR analysis of EerI-p97 interaction. Representative binding curves show direct binding of EerI to p97. R.U. response unit. [file pone.0015479.s001.tif]

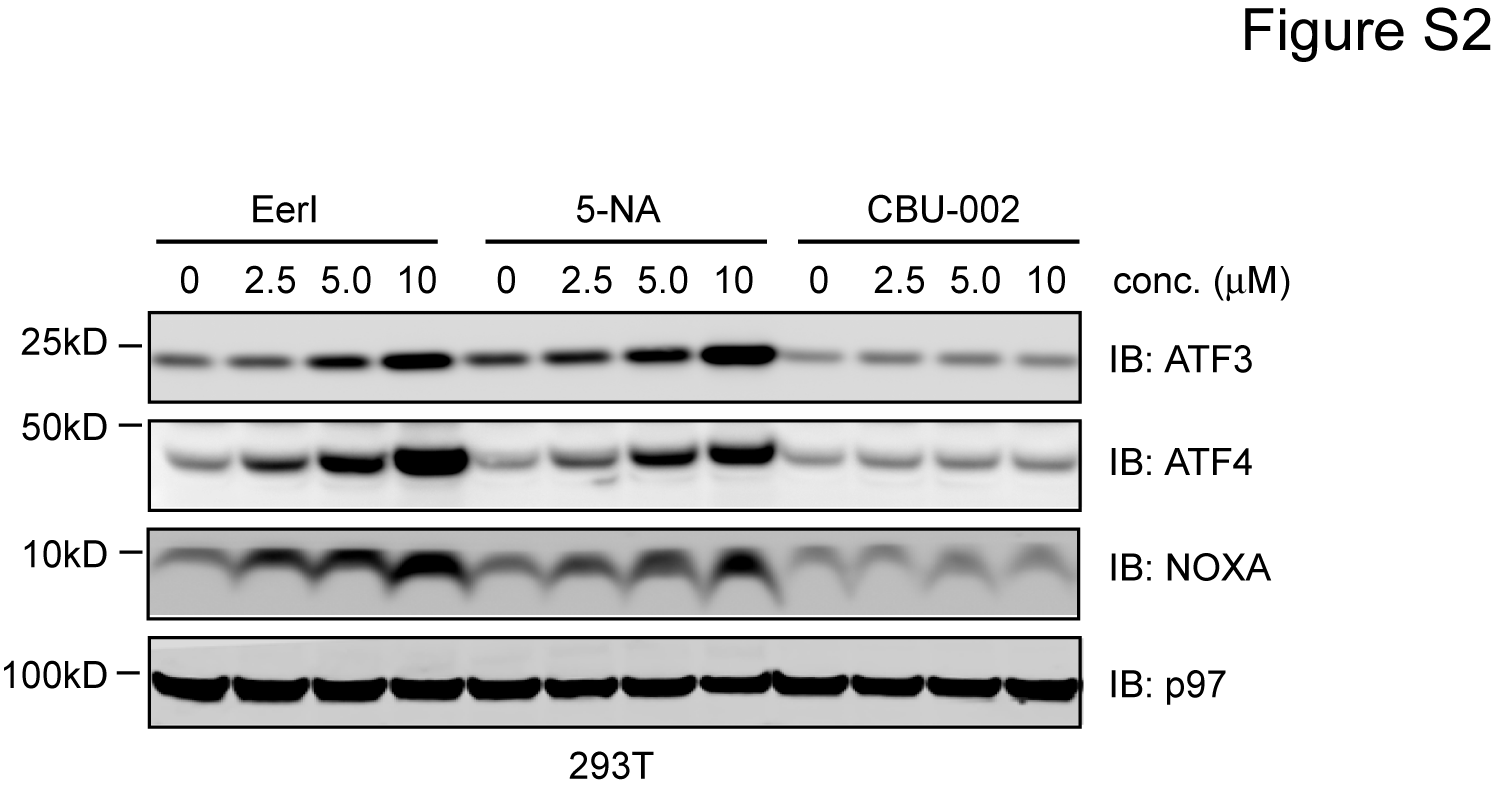

Supplement: Figure S2 — Induction of ER stress and NOXA expression by EerI and 5-NA. 293T cells were treated with various compounds at the indicated concentration for 8 h. Whole cell extract was analyzed by immunoblotting with the indicated antibodies. [file pone.0015479.s002.tif]

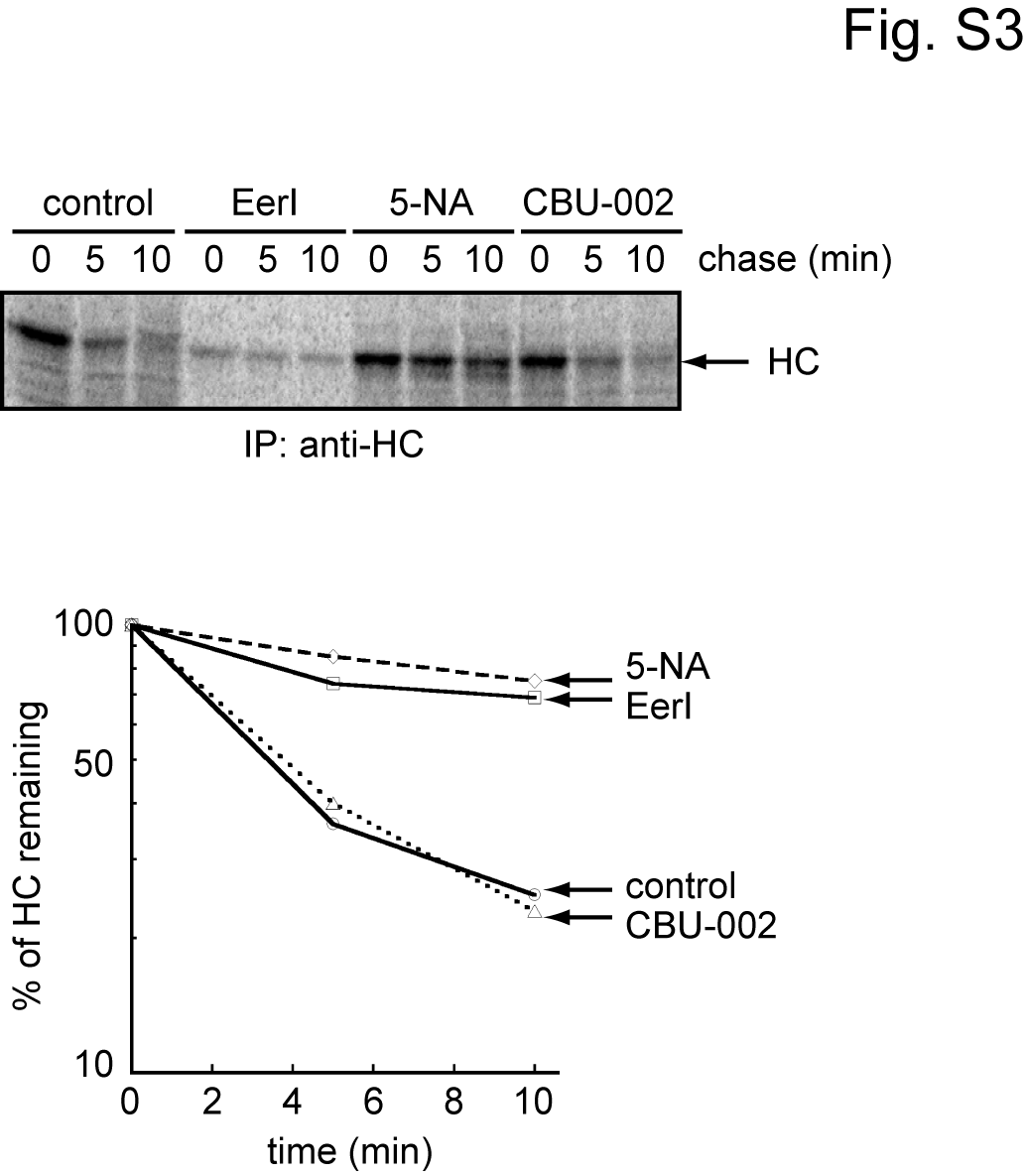

Supplement: Figure S3 — 5-NA but not CBU-002 inhibits the degradation of MHC class I heavy chain in US11 cells. US11 cells treated with the indicated compound (10 µM) were pulse labeled in a medium containing 35S-Met/Cyc then incubated in a chase medium containing excess unlabeled Met/Cys. MHC class I heavy chain was immunoprecipitated from cell extract and analyzed by SDS–PAGE and autoradiography. [file pone.0015479.s003.tif]

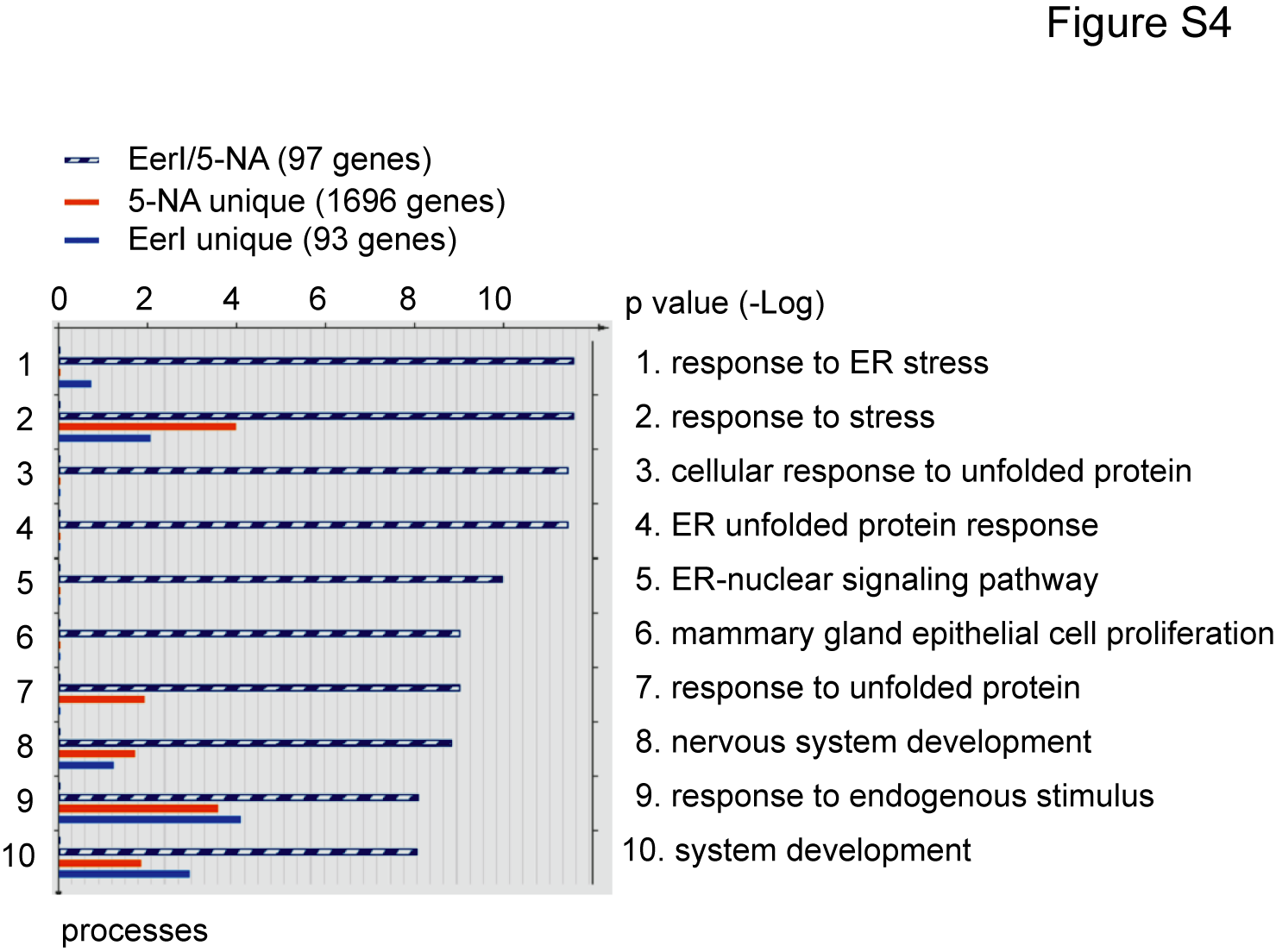

Supplement: Figure S4 — Pathway analyses of EerI and 5-NA signature genes. Genes with fold change ≥1.5 from ANOVA analysis (p value ≤0.05) were used for pathway analyses by the commercial software MetaCore. Shown is a histogram indicating the top 10 cellular processes significantly represented by genes affected by both Eer1 and 5-NA. For comparison, the relative representation of the corresponding pathway by the unique genes for 5-NA or Eer1 was shown in red and in blue, respectively. [file pone.0015479.s004.tif]
