# Supplementary material for: The ERAD Inhibitor Eeyarestatin I Is a Bifunctional Compound with a Membrane-Binding Domain and a p97/VCP Inhibitory Group
Source: PLoS One. 2010 Nov 12;5(11):e15479. doi: 10.1371/journal.pone.0015479 (PMC2993181; doi:10.1371/journal.pone.0015479)
Supplement: Table S2 — A list of compounds tested in this study. JEKO-1 cells were treated with each of the listed compounds at concentrations ranging from 1.25 µM to 20 µM. Cell viability was determined by the MTT assay and used to obtain concentration-response curve. IC50 was extracted by linear interpolation. [file pone.0015479.s006.doc]

**Supplementary table 2**

| **S. No.** | **Expt. No.** | **Structure** | **IC 50 values (μM)** |
| --- | --- | --- | --- |
| CBU-001 | BAS-08-134 |  | >40 |
| CBU-002 | BAS-08-135c |  | >40 |
| CBU-003  [**EER1**] | BAS-08-136 |  | 3.5 |
| CBU-004 | BAS-08-137 |  | >40 |
| CBU-005 | BAS-08-138b |  | >40 |
| CBU-006 | BAS-08-139 |  | >40 |
| CBU-007 | BAS-08-140 |  | 30 |
| CBU-008 | BAS-08-141 |  | 2.5 |
| CBU-009 | BAS-08-142 |  | >40 |
| CBU-010 | BAS-08-143 |  | >40 |
| CBU-011 | BAS-08-144 |  | >40 |
| CBU-012 | BAS-08-146 |  | >40 |
| CBU-013 | BAS-08-148 |  | 3.0 |
| CBU-014 | BAS-08-149 |  | 3.0 |
| CBU-015 | BAS-08-186 |  | 8.0 |
| CBU-016 | BAS-08-187 |  | 7.0 |
| CBU-017 | BAS-08-188 |  | 15.0 |
| CBU-018 | BAS-08-189 |  | 18.0 |
| CBU-019 | BAS-08-190 |  | 3.5 |
| CBU-020 | BAS-08-191 |  | 4.0 |
| CBU-021 | BAS-08-200 |  | 8.0 |
| CBU-022 | BAS-08-204 |  | 6.0 |
| CBU-023 | BAS-08-206 |  | 15.0 |
| CBU-024 | BAS-08-207 |  | 7.0 |
| CBU-025 | BAS-08-208 |  | 6.5 |
| CBU-026 | BAS-08-209 |  | 12.0 |
| CBU-027 | BAS-08-210 |  | 7.5 |
| CBU-028  [**211**] | BAS-08-211 |  | 3.0 |
| CBU-029 | BAS-08-214 |  | >40 |
| CBU-030 | BAS-08-219 |  | >40 |
| CBU-031 | BAS-08-224 |  | >40 |
| CBU-032 | BAS-08-231 |  | 25.0 |
| CBU-033 | BAS-08-232 |  | 6.0 |
| CBU-034 | BAS-08-233 |  | 20.0 |
| CBU-035 | BAS-08-234 |  | >40 |
| CBU-036 | BAS-08-235 |  | 9.0 |
| CBU-037 | BAS-08-236 |  | >40 |
| CBU-038 | BAS-08-237 |  | 9.0 |
| CBU-039 | BAS-08-238 |  | 15.0 |
| CBU-040 | BAS-08-239 |  | 10.0 |
| CBU-041 | BAS-08-240 |  | 10.0 |
| CBU-042 | BAS-08-241 |  | 40.0 |
| CBU-043 | BAS-08-242 |  | 30.0 |
| CBU-044 | BAS-08-243 |  | >40 |
| CBU-045 | BAS-08-244 |  | 6.0 |
| CBU-046 | BAS-08-245 |  | 10.0 |
| CBU-047 | BAS-08-246 |  | 30.0 |
| CBU-048 | BAS-08-251 |  | 30.0 |
| CBU-050 | BAS-09-6 |  | >40 |
| CBU-051 | BAS-09-37 |  | 10.0 |
| CBU-052 | BAS-09-38 |  | >40 |
| CBU-053 | BAS-09-39 |  | >40 |
| CBU-054 | BAS-09-40 |  | 15.0 |
| CBU-055 | BAS-09-41 |  | 6.0 |
| CBU-056 | BAS-09-42 |  | >40 |
| CBU-057 | BAS-09-43 |  | >40 |
| CBU-058 | BAS-09-44 |  | >40 |
| CBU-059 | BAS-09-45 |  | >40 |
| CBU-060 | BAS-09-46 |  | >40 |
| CBU-061 | BAS-09-47 |  | 5.0 |
| CBU-062 | BAS-09-48 |  | 2.5 |
| CBU-063 | BAS-09-50 |  | >40 |
| CBU-064 | BAS-09-51 |  | >40 |
| CBU-065 | BAS-09-54 |  | >40 |
| CBU-066 | BAS-09-55 |  | >40 |
| CBU-067 | BAS-09-56 |  | >40 |
| CBU-068 | BAS-09-57 |  | >40 |
| CBU-069 | BAS-09-58 |  | >40 |
| CBU-070 | BAS-09-59 |  | >40 |
| CBU-071 | BAS-09-60 |  | >40 |
| CBU-072 | BAS-09-61 |  | >40 |
| CBU-073 | BAS-09-101 |  | 3.0 |
